# Combination of surgical excision and custom designed silicon pressure splint therapy for keloids on the helical rim

**DOI:** 10.1186/1746-160X-3-14

**Published:** 2007-03-12

**Authors:** Michael Sand, Daniel Sand, Pejman Boorboor, Benno Mann, Peter Altmeyer, Klaus Hoffmann, Falk G Bechara

**Affiliations:** 1 Department of General and Visceral Surgery, Augusta Kranken Anstalt, Academic Teaching Hospital of the Ruhr-University Bochum, Germany; 2 Department of Physiological Science, University of California Los Angeles (UCLA), Los Angeles, California, USA; 3 Department of Plastic and Reconstructive Surgery, Hannover Medical School, Hannover, Germany; 4 Department of Dermatology and Allergology, Ruhr-University Bochum, Germany

## Abstract

Keloids are defined as dermal fibrotic lesions which are considered an aberration of the wound healing process. Their etiology and pathogenesis are poorly understood. Different treatment modalities are described in the literature depending on the morphology and size of the keloid. We report a case of a large ear keloid on the helical rim which was successfully treated with surgery and a custom designed silicon pressure clip.

## Background

Keloids are defined as dermal fibrotic lesions which are considered an aberration of the wound healing process. They are included in the spectrum of fibroproliferative disorders and can potentially occur anywhere on the body. Areas more commonly affected are the anterior chest, shoulders, flexor surfaces of the extremities, and the ears.

Keloids on the ears present several therapeutic challenges. They are common after small skin excisions and other procedures, including drainage of auricular hematomas, repair of other auricular traumas, or as secondary keloid formation after prior keloid excision.

Several procedures have been described for effective treatment of keloid scars. They include silicon occlusive dressings, mechanical compression, radiation, cryosurgery, topical Imiquimod application, bleomycin tattooing, intralesional injections of steroids, 5-floururacil, as well as interferon-alpha, -beta or -gamma in combination with excisional surgery [1-7]. Although optimal conditions for the prevention of keloid formation are still unknown the combination of exicisional surgery and the placement of a silicone gel sheet over the wound surface with the application of light pressure are known to be advantageous [8-10].

In the following case report we describe a custom designed silicon pressure splint which was successfully used for preventive, postoperative treatment of a large keloid formation on the helical rim.

## Case

**Figure 1:**
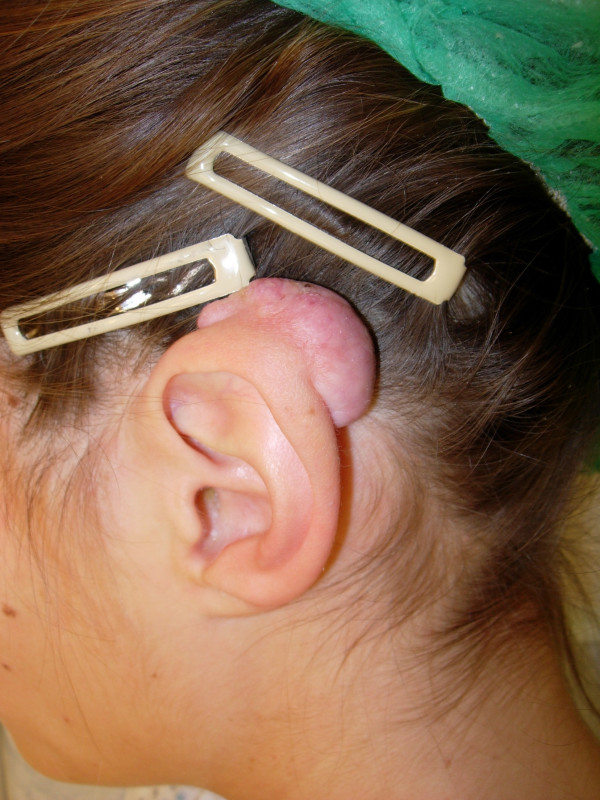
Plum-sized keloid on the left helical rim.

**Figure 2:**
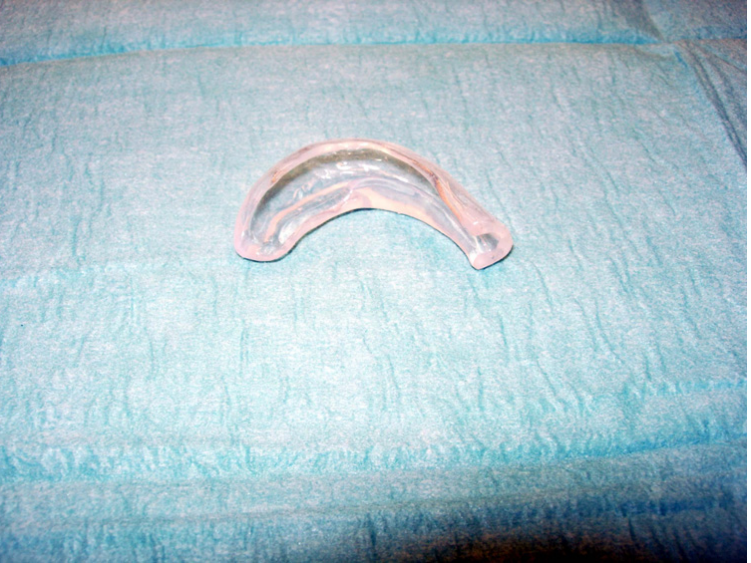
Custom-build silicon pressure splint for the left helical rim.

A 25-year-old Caucasian female with skin type 2 (Fitzpatrick classification) presented because of a plum-sized pedunculated keloid on the upper part of her left helical rim. She reported that 10 years ago she had already experienced formation of a nodule in this area which became evident 6 months after an ear piercing. This keloid-like nodule was excised twice and injected with steroids. At the time of presentation the plum-sized keloid on her helical rim had been increasing in size and was accompanied by severe pruritus (Fig 1). We introduced our patient to an audiology technician in order to design and build a specially silicon pressure splint for her left ear (Fig 2).

**Figure 3:**
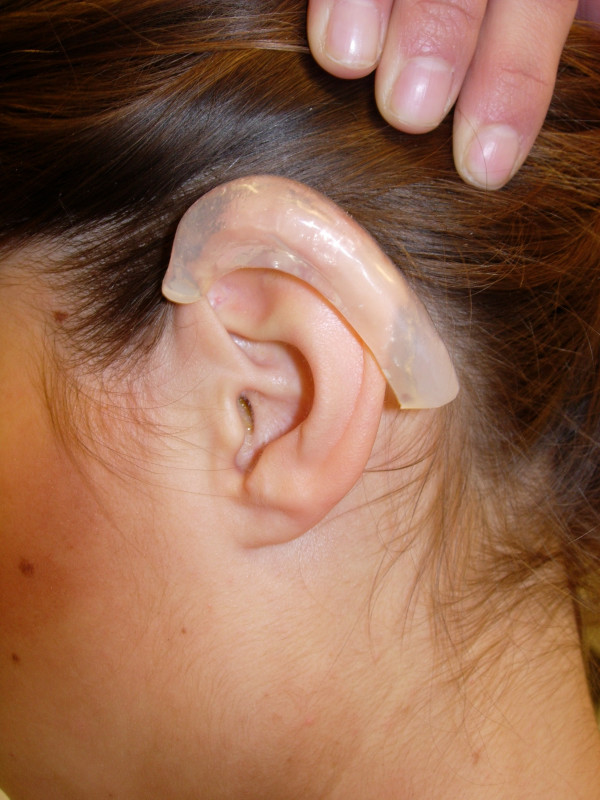
Patients' left ear after keloid excision with silicon pressure splint on the left helical rim.

**Figure 4:**
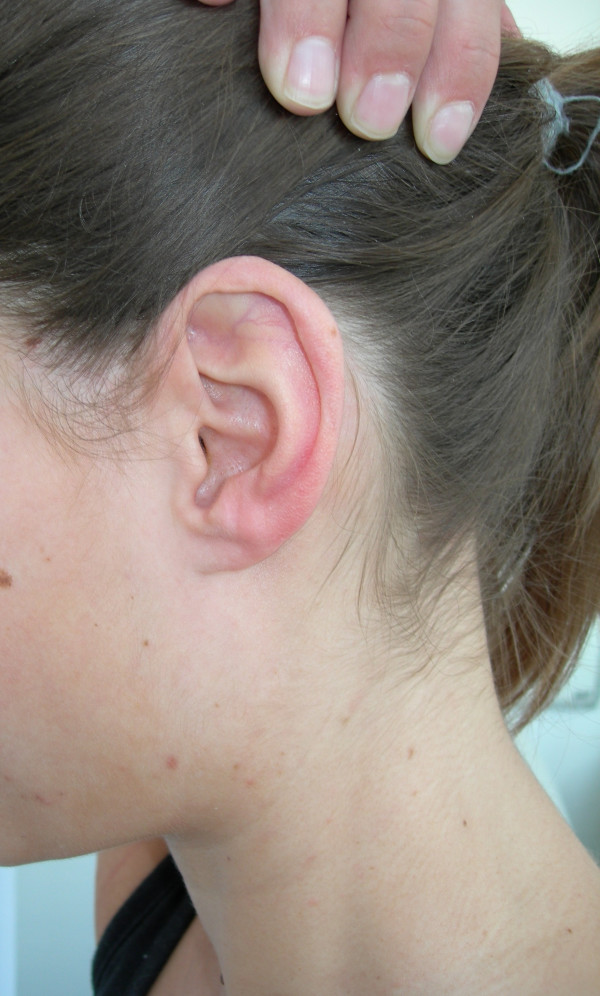
Lateral view on the patients left ear 24 months after the last injection.

**Figure 5:**
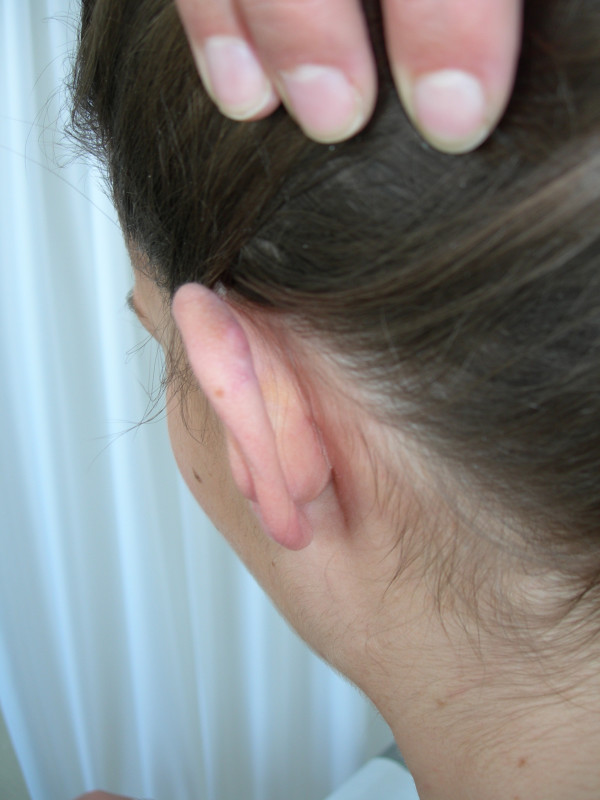
Posterior view on the patients left ear 24 months after the last injection.

The keloid was then excised with cold steel. Immediately after the operation a combination of 0.5 ml triamcinolonacetonid and scandicain 2 % was intralesionally injected. The custom made silicon splint was applied directly after surgery and steroid injection (Fig 3). The injections were repeated at intervals of 8 weeks for 12 months. The patient was instructed to wear the splint for 24 h a day, 7 days a week. A clinical check-up one year and 24 months after the last injection showed no tendency to relapse (Fig 4 and Fig 5).

## Discussion

Although the incidence of keloid formation is predominantly in darkly pigmented individuals, who form keloids up to 19 times more than Caucasians, those Caucasians who do are among skin types I and II and, as in our patient, are the most difficult ones to treat [11]. After an earring piercing, our patient experienced the third relapse of a keloid on the helical rim which had been unsuccessfully treated with excision and intralesional steroid-injection before.

After surgery, the combination of several preventive steps is essential for a successful treatment plan. It is known that surgical monotherapy results in a high incidence of recurrence (50–100%) [12,13]. Additionally surgical excision and primary closure should be performed with as little wound tension as possible which is not always an easy task where the amount of skin is limited, as on the anterior side of the ear. Hence, we utilized multi-modal standard therapy forms in this patient.

Surgical excision and postoperative intralesional injection of steroid was combined with silicon gel sheeting and compression therapy with an individually designed silicon pressure splint for the helical rim. The procedure combines the advantageous effects of pressure and silicon gel sheeting. Silicon has been described as effective in preventing the development of keloids. It reduces keloid scar formation by 70% when used consistently [14]. There are several theories of the action mechanism. Although some authors propose that silicon diffuses from the surface of the silicon gel sheets and reduces keloid ground substance it is more likely that retardation of epidermal water loss and a subsequent increase of wound hydration is responsible for the keloid-inhibiting [15,16].

Compression therapy with dressings or devices that apply more than 24 mmHg, the capillary pressure, create a hypoxic microenvironment which results in fibroblast, and, subsequently, collagen degradation. Pressure earrings with compression plates which are available in different sizes are successfully used for ear lobe keloids. It is obvious that the helical rim with its concave anterior and convex posterior surface is not easily amenable for compression. The silicon pressure splint introduced here not only enjoys all the advantages of silicon dressings but also successfully delivers pressure on the helical rim.

We suggest that in cases of keloids on the helical rim the above described custom designed silicon pressure splint combined with subsequent steroid injections respects the delicate anatomy of the helical rim and can be a therapeutic approach with strong benefit for the patient.

## Authors' contributions

MS: Surgeon who performed the operation, documented and prepared the draft

DS: Literature search, revision of bibliography and helped with editing of the manuscript     

PB: Helped in preparing the draft    

BM: Edited most of the manuscript    

PA: Revised and edited the manuscript and helped in preparing the draft     

KH: Literature search and edited part of the manuscript     

FGB: Surgeon who performed the operation and edited part of the manuscript and helped in preparing the draft     
